# A growing toolbox of techniques for studying β-barrel outer membrane protein folding and biogenesis

**DOI:** 10.1042/BST20160020

**Published:** 2016-06-09

**Authors:** Jim E. Horne, Sheena E. Radford

**Affiliations:** * Astbury Centre for Structural Molecular Biology and School of Molecular and Cellular Biology, The University of Leeds, Leeds LS2 9JT, U.K.

**Keywords:** β-barrel, biogenesis, biophysical techniques, outer membrane protein, protein folding

## Abstract

Great strides into understanding protein folding have been made since the seminal work of Anfinsen over 40 years ago, but progress in the study of membrane protein folding has lagged behind that of their water soluble counterparts. Researchers in these fields continue to turn to more advanced techniques such as NMR, mass spectrometry, molecular dynamics (MD) and single molecule methods to interrogate how proteins fold. Our understanding of β-barrel outer membrane protein (OMP) folding has benefited from these advances in the last decade. This class of proteins must traverse the periplasm and then insert into an asymmetric lipid membrane in the absence of a chemical energy source. In this review we discuss old, new and emerging techniques used to examine the process of OMP folding and biogenesis *in vitro* and describe some of the insights and new questions these techniques have revealed.

## Introduction

**Figure 1:**
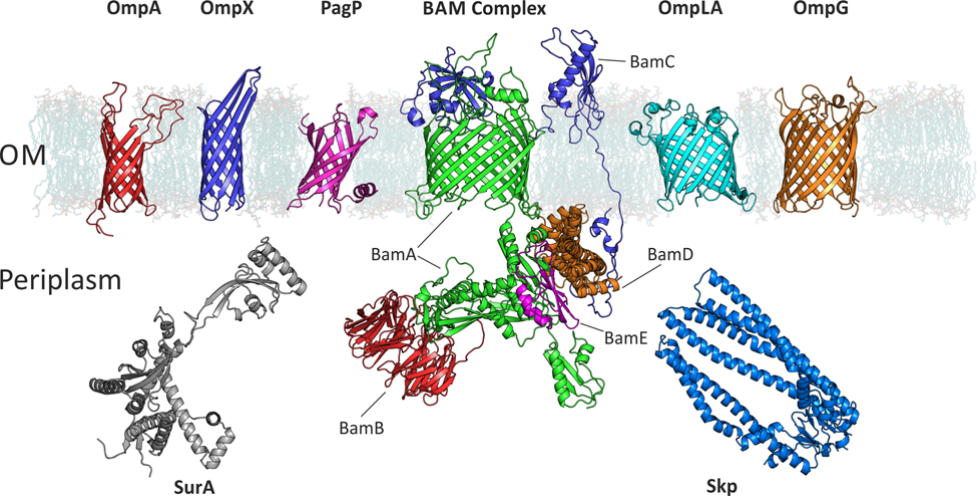
Examples of OMPs and chaperones mentioned in this review BAM complex: BamA–green, BamB–red, BamC–blue, BamD–orange, BamE–magenta. PDB ID of structures: OmpA (1G90); OmpX (1QJ8); PagP (1THQ); BamABCDE ([21]); OmpLA (1QD6); OmpG (2IWW); SurA (1M5Y, missing regions built using MODELLER); Skp (1U2M, missing regions built using PyMol). DMPC membrane from O'Neil et al. [21].

**Figure 2:**
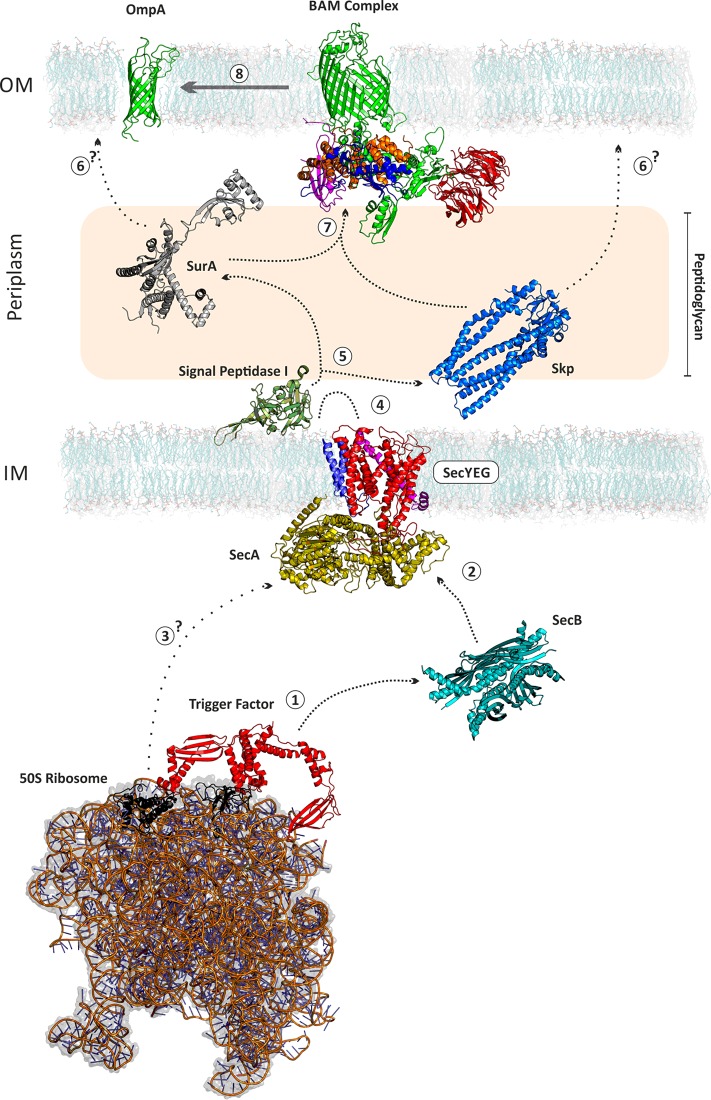
Biogenesis of OMPs A nascent OMP emerges from the ribosome and is bound by trigger factor (1) before being passed to SecA via SecB (2), alternatively nascent chains may interact directly with SecA (3). The unfolded OMP (uOMP) passes through the SecYEG channel and the signal sequence is inserted into the inner membrane (IM) (4). This sequence is cleaved by signal peptidase I and the uOMP is bound by the chaperones Skp and/or SurA (5). The uOMP can then be delivered directly to the outer membrane (OM) (6) or to the BAM complex (7). The BAM complex then catalyses the OMP's folding into the OM (8). SecYEG complex: SecY–red, SecE–magenta, SecG–blue, SecA–yellow. BAM complex: BamA–green, BamB–red, BamC–blue, BamD–orange, BamE–magenta. All proteins are shown to scale. The length of the periplasmic space from leaflet to leaflet is scaled to 180 Å. PDB ID of structures: OmpA (1G90); BamACDE (5EKQ); BamB (4XGA); SurA (1M5Y, missing regions built using MODELLER); Skp (1U2M, missing regions built using PyMol); signal peptidase I (1KN9); SecYEG+SecA (3DIN); SecB (1OZB); Trigger Factor (3GU0); 50S ribosome (2D3O). DMPC membrane from O'Neil et al. [21].

The study of protein folding underpins a goal to understand the function of biological systems in terms of the structures, properties and interactions of the molecules which orchestrate many of life's essential processes. The field of protein folding sits at an intersection between scientific disciplines and requires a plethora of complementary techniques to be combined to answer the question “How do proteins fold?” Although many of the techniques and underlying principles learned from over 40 years of studies on the folding of water soluble proteins [1,2] can be applied to membrane proteins, the introduction of the lipid bilayer and its steric and physicochemical properties necessarily alters the forces that guide protein folding when coupled with insertion into the bilayer itself. The outer membranes (OM) of mitochondria, chloroplasts and Gram-negative bacteria consist almost entirely of β-barrel outer membrane proteins (OMPs) (Figure 1). The assembly of OMPs has received significant attention in the last few years after the discovery of an essential protein machinery, the β-barrel assembly machine complex (or BAM complex), which is required for the assembly of OMPs into the OM of Gram-negative bacteria (Figure 2) [3–6]. The OM provides a fundamentally different folding environment compared with the inner membrane: the bilayer is asymmetric as it is enriched in lipopolysaccharide in the outer leaflet, it is densely packed with OMPs, and diffusion is restricted [7,8].

Early studies of OMP folding focused on obtaining an understanding of folding/unfolding rates and equilibria and the conditions that alter them for a small set of OMPs [9–14]. Most recently, however, application of modern biophysical techniques is allowing more challenging mechanistic questions about OMP folding to be tackled, as highlighted below.

## ‘Classic’ methods of interrogating folding applied to β-barrel membrane proteins

### Gel assays

**Figure 3:**
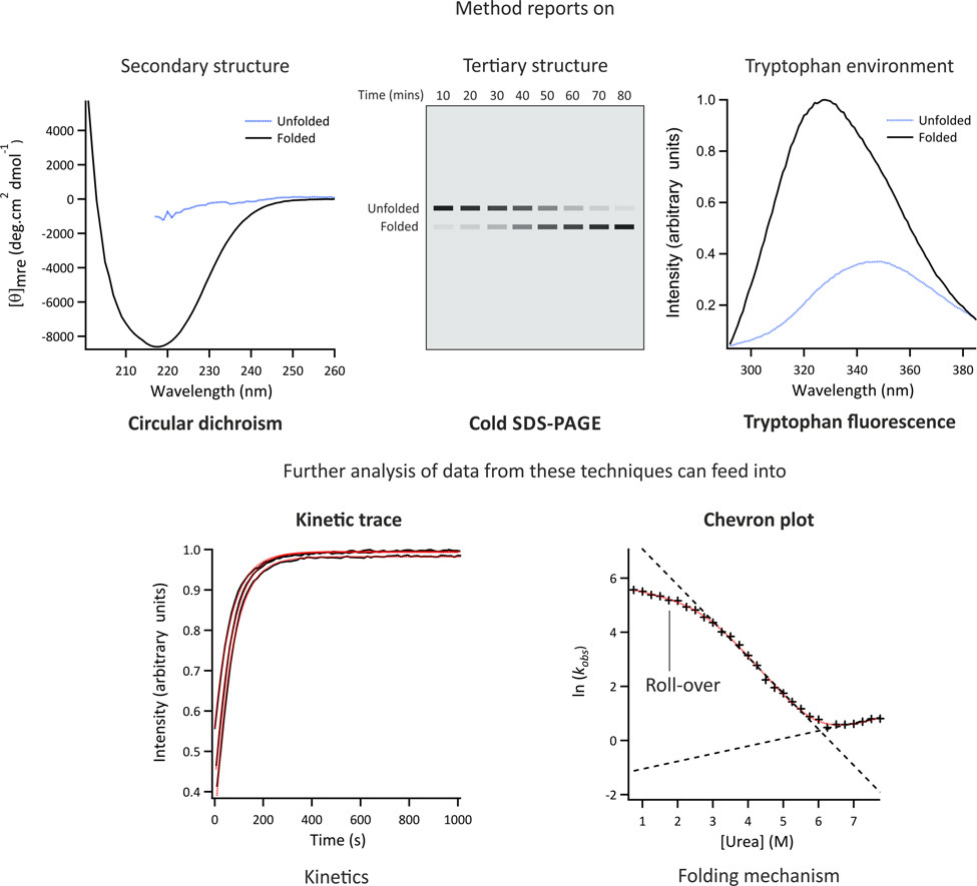
‘Classic’ methods of interrogating protein folding Different techniques provide independent, and complementary, information about the kinetics, thermodynamics and mechanism of folding. These approaches have been used for analysis of water soluble and OMP folding (see text). *CD* reports on the difference in absorbance of left and right circularly polarized light by peptide bonds in an asymmetric environment. In this case, the asymmetric environment refers to the protein's secondary structure, with e.g. β-sheet, α-helix and disorder each giving rise to characteristic spectra. CD in the far UV can be used for the analysis of both water soluble proteins and OMPs. [*θ*]_mre_; mean residue ellipticity. *Cold SDS-PAGE* is useful for the analysis of the formation of native OMPs since these structures are resistant to denaturation in SDS without heating. This method involves initiating a folding reaction, taking samples at particular time points and quenching further folding with SDS, then running the samples on an SDS-PAGE gel *without* boiling. These gels can be analysed qualitatively or quantitatively by measuring the band intensity through densitometry. *Tryptophan fluorescence* is sensitive to the polarity of the local environment. Upon folding, tryptophan residues which move to more hydrophobic environments (such as within a folded protein core, or interfaced with lipid membranes or detergents) show a characteristic ‘red-shift’ in their emission maxima and, usually, an increase in fluorescence intensity. *Kinetic traces* can be obtained by monitoring the change in signal intensity from CD and/or tryptophan fluorescence at particular wavelengths or, for OMPs, by analysing samples taken at different refolding times using cold SDS-PAGE. *Chevron plots* involve measuring the rate constants for folding/unfolding from kinetic traces measured in increasing concentrations of denaturant and plotting the natural logarithm of these values as a function of denaturant concentration. Red lines indicate calculated fits.

Cold SDS-PAGE exploits the observation that many OMPs are resistant to SDS denaturation and so the unfolded and folded states migrate differently on a gel when loaded without boiling (Figure 3, *top centre*) [9,15–17]. This approach can be used to determine the fraction of OMPs folded at certain time points (e.g. following initiation of folding from a urea-denatured state) and thereby to extract rate constants of folding [18]. Gel assays were used to examine the effect of membrane thickness on folding yields into large unilamellar vesicles (LUVs) formed from didecanoylphosphatidylcholine (DDPC, *di*C_10_PC) to dioleoylphosphatidylcholine (DOPC, *di*C_18:1_PC). For most OMPs decreasing lipid chain length increases the folding yield, while folding into DOPC LUVs is almost completely abrogated [17,19]. Comparative studies with OmpA showed a close correlation between the results obtained using gel assays and tryptophan fluorescence [20].

### Circular dichroism (CD)

Far-UV CD enables the formation of secondary structure to be monitored (Figure 3, *top left*). The influence of the lipid phase on folding and insertion of the OmpA β-barrel into LUVs composed of dimyristoylphosphatidylcholine (DMPC, *di*C_14_PC) or *di*C_13_PC was tested by following the change in CD at 216 nm. The authors found that the folding rates increase when the bilayer is at its transition temperature (*T*_m_) [22]. Studies of OmpG folding into octyl glucoside (OG) showed a ‘burst-phase’ of β-sheet formation followed by a second phase in which a native-like content of secondary structure forms with a *t*_1/2_ of minutes [23]. Gel assays, however, showed that formation of the native state occurs on a timescale of hours. Together this indicated that hydrophobic collapse and/or adsorption to micelles and formation of secondary structure represent intermediate steps preceding formation of the native state and closing of the tertiary β-barrel of OmpG. Conversely, a more concerted picture of coupled secondary and tertiary structure formation was observed using similar methods to follow the folding of OmpA in DMPC or 95:5 DMPC/dimyristoylphosphatidylglycerol (DMPG) small unilamellar vesicles (SUVs) [24], as well as DDPC, diundecanoylphosphatidylcholine (DUPC, *di*C_11_PC), dilauroylphosphatidylcholine (DLPC, *di*C_12:0_PC) or DMPC LUVs [19]. Early formation of secondary structure elements may reflect the formation of misfolded or off-pathway intermediates as a consequence of the rapid collapse of these membrane proteins in the aqueous phase prior to membrane insertion [24,25]. Further experiments will be needed using different OMPs and different folding conditions to determine whether this is the case or not for different proteins and different lipid environments.

### Tryptophan fluorescence

All OMPs characterized to date contain aromatic residues (commonly tryptophan) that form a girdle at the bilayer:aqueous interface and are thought to be important in stabilizing OMPs within the membrane. The fluorescence signal of these Trp residues provides a useful probe of the folding status of OMPs (Figure 3, *top right*) [26]. By following fluorescence intensity compared with time, Trp fluorescence has been used to measure the kinetics of OMP folding/unfolding and to derive their folding/unfolding free energies (Figure 3, *lower left*) [27]. Studies following the change in the centre of spectral mass for Trp emission of OmpA folding into vesicles of different sizes at different concentrations of urea, found an influence of stored membrane curvature elastic stresses on the transition state of folding [28].

### Quenching of tryptophan fluorescence

‘Time-resolved distance determination by fluorescence quenching’ (TDFQ) involves the incorporation of a bromine atom into lipids at specific positions [29]. The bromine atom quenches tryptophan emission with an *R*_0_ (the distance of 50% quenching efficiency) of around 9 Å (1 Å=0.1 nm) [30]. OmpA contains five tryptophan residues, one on the periplasmic side of the barrel and the other four on the extracellular side, with one in each β-hairpin loop. Using this methodology the authors were able to trap a number of intermediates during the folding of OmpA into DOPC SUVs [29]. Using site-directed mutagenesis to create single-Trp variants the authors found that all four hairpins cross the bilayer concurrently, suggesting a model in which insertion is directly coupled to folding of the β-barrel domain [31]. More detail on the mechanism and order of insertion and β-strand association was obtained through *intra*molecular quenching of single-Trp variants of OmpA in the same *in vitro* system by a nitroxyl spin-label (which quenches Trp fluorescence at distances <10–20 Å [32,33]) conjugated to a mutant cysteine in the neighbouring strand. The results suggested that association of the N- and C-terminal strands may occur in tandem with membrane adsorption (i.e. early, not late in the folding process) and that residues in the extracellular regions of pairs of β-strands in the β-barrel associate before those in the periplasmic ends of the β-strands [34].

### Chevron plots

Plots of the natural logarithm of observed rate constants for folding/unfolding against denaturant concentration are commonly used to analyse the folding pathways of water soluble proteins (Figure 3) [35]. This approach was used to interrogate the folding mechanism of PagP (Figure 1) into DLPC LUVs [36]. The results indicated a reversible two-state folding mechanism for PagP under the concentration range of denaturant used involving a transition state that is 50% as compact as the native protein [36]. By contrast, studies of OmpA folding in guanidinium chloride showed that at low denaturant concentrations the linear folding phase of the chevron plot becomes non-linear–a phenomenon termed ‘roll-over’ (Figure 3, lower right) which suggests a three-state folding mechanism [37].

### *φ*-value analysis

*φ*-value analysis is a powerful technique for acquiring information on the structure and stability of non-native states formed during protein folding. Originally developed for soluble proteins [38,39], this approach has been applied only recently to membrane proteins [36,40]. It involves making mutations in specific residues of a protein, measuring the change in activation energy for unfolding (ΔΔ*G*°_N − trans_, the free energy required to overcome the transition state barrier) and the equilibrium free energy of unfolding (ΔΔ*G*°_U_), and comparing the ratio between the two to derive a *φ*-value. A value of 1 implies that native structure has already formed in the transition state *at that particular residue*, and a value of 0 that native structure has not yet formed. Analysis of the transition state for PagP folding/unfolding showed that the β-barrel is largely formed at this stage, that insertion may occur via a ‘tilted’ orientation and that the N-terminal α-helix (Figure 1) assembles late in the folding pathway [36].

## Augmenting our understanding of folding with advanced biophysical techniques

### Structural methods for the analysis of membrane proteins

Knowing the native structure of a protein is vital in order to interpret information about its folding mechanism. Large and dynamic proteins which require a lipid membrane for solubility present challenges for structural studies but new methodologies are emerging for acquiring structural information.

### Mass spectrometry (MS)

Until recently, mass spectrometry was limited to analysis of peptides or water soluble proteins, but recent developments have enabled previously intractable membrane protein complexes to be analysed from detergent micelles or nanodiscs using MS [41,42]. Other membrane mimetics (such as amphipols) have also been developed [43–45], and functional and structural studies on OMPs using MS are beginning to be reported [46]. Full-length OmpA has been studied in depth using native MS and ion-mobility spectrometry–MS (IMS–MS). The results revealed a dimer interface between the periplasmic domains [47] and, along with cross-linking experiments [48], have been used to build models of the structure of full length OmpA for the first time.

### NMR

NMR is a powerful tool for elucidating protein folding mechanisms [49]. For water soluble proteins, NMR has revealed insights into the nature of unfolded and intrinsically disordered states of proteins, as well as partially folded intermediates and even rare (1%) populated partially structured states [50–52]. ^1^H–^1^H NOEs along with a number of complementary NMR experiments have been used to show that in 8 M urea OmpX is globally denatured, but contains locally structured regions [53]. These locally structured regions are formed in an area of hydrophobic clustering around a tryptophan residue which may be relevant for early intermediate stages of folding involving membrane adsorption [31,54]. NMR studies of chaperone:unfolded-OMP complexes have proved particularly fruitful in elucidating the mechanism of chaperoning in the periplasm and the conformation of OMPs in their chaperone-bound states [55]. Studies have shown that the chaperone Skp binds unfolded OMPs in a compact unfolded state via hydrophobic, low affinity, high avidity interactions within the internal cavity of Skp (Figure 1) [56,57]. This may indicate a requirement for folding to proceed from a high entropy, low enthalpy unfolded state.

### Computational methods

#### MD

MD simulations allow atomic-level detail to be obtained about protein folding that is not usually accessible experimentally. The level of computing power now available and improvements in force fields are opening up new avenues for research, with reported simulations now passing the millisecond time-scale [58,59]. Models of DLPC and simulated OM bilayers have been constructed and the *in silico* behaviour of OmpLA in these membranes assessed [60,61]. This system has been used to show that OmpLA causes local thinning of the bilayer due to hydrophobic mismatch, and that lipopolysaccharide in the outer leaflet stabilizes OmpLA's extracellular loops [60,61]. Course-grained models of membranes have been used to study the *insertion* of ‘pre-folded’ OmpA, revealing that the β-barrel perturbs the bilayer structure and inserts at a 45° angle before equilibrating to an orientation parallel to the bilayer normal [62]. This tilted-insertion mechanism is consistent with *in vitro* experiments on PagP [36], although more examples need to be gathered to determine whether this is a general mechanism for OMP folding.

#### Bioinformatics

One open question regarding OMP biogenesis is how the different folding chaperones and catalysts in the periplasm are able to recognize OMPs compared with soluble periplasmic proteins and assist OMP delivery to the BAM complex (Figure 2). The current consensus is that a C-terminal aromatic-rich sequence (β-signal) found in all OMPs is key for this process [63–65]. Bioinformatic analysis of the entire predicted OMP proteome from 437 bacterial strains showed that the β-signal motifs are highly conserved, with notable variants in *Helicobacter* and *Neisseria* spp. [66]. This emphasizes the significance of the β-signal of OMPs for their biogenesis. Future comparative analysis of the co-evolution of outlier species’ OMPs with their periplasmic folding machinery may allow us to identify the regions of chaperones and their assembly machinery that are tailored to recognize this signal.

### Single molecule methods

#### Force microscopy

Single molecule force spectroscopy has been used to analyse the unfolding of proteins by application of a stretching force and measurement of the protein's resistance to deformation [67]. Such studies have shown that OmpG unfolds via a series of intermediates, corresponding to two β-strands (a β-hairpin) unfolding at a time [68]. The interaction strengths stabilizing β-strands in OMPs (150–250 pN) were found to be around 1.5 times greater than those of α-helical membrane proteins (100–150 pN) [68]. A recent elegant study analysed the influence of the periplasmic ATP-independent chaperones, Skp and SurA, on the refolding of the 22-stranded OMP, FhuA (Figure 1) [69]. The authors found that in the absence of chaperones, FhuA misfolded or remained unfolded in 93% of the experiments. Addition of Skp resulted in FhuA being trapped in an unfolded state. By contrast, SurA reduced misfolding, but also increased the probability of successful folding events (to 40%). The results give insight into how OMP biogenesis may proceed *in vivo* and promotes the idea of the membrane as a free energy sink into which unfolded OMPs insert and fold [70].

#### Single-molecule tracking (SMT)

Single-molecule tracking (SMT) microscopy can follow the fate of individual proteins *in vivo* and *in vitro* and is beginning to emerge in the study of OMPs [7]. SMT total internal reflection fluorescence microscopy (SMT-TIRFM) was used to show that OMPs cluster in highly dense and diffusionally restricted ‘islands’ *in vivo* that co-localize with the BAM complex [71]. *In vitro* it was also shown that even OMPs at ‘uncrowded’ lipid:protein ratios (100,000:1) self-associate over time such that their diffusion within a membrane slows [71]. These data add to an increasingly large literature suggesting the membrane environment into which OMPs must fold *in vivo* is crowded and diffusionally restricted [7].

### Förster energy transfer (FRET)

FRET occurs when there is radiationless transfer of energy from a fluorescent donor to an acceptor which may itself fluoresce, or quench the fluorescence of the donor [72,73]. FRET can be used as a sensitive probe of inter- and/or intra-molecular distances. Changes to the oligomerization state of OmpLA were studied *in vitro* by monitoring the association of two populations of donor and acceptor labelled protein at putative dimerization sites upon introduction of a calcium ion [74]. The folding of the OmpA β-barrel has also been probed by FRET using a single-tryptophan mutant as donor and 1,5-IAEDANS covalently attached to a cysteine residue as acceptor [75]. This study showed the feasibility of using FRET to study OMP folding, suggesting that there is early formation of a pore-like structure prior to traversal of the membrane, and that later steps may involve extension of β-strands and opening of the pore [75].

## Conclusions

New technologies and methodologies are making experimentally intractable questions about membrane protein folding amenable for analysis through advanced biophysical techniques. When used in combination with ‘classical’ techniques for studying protein folding they can reveal previously unseen levels of detail in the mechanisms by which OMPs fold. Single molecule techniques can distinguish whether OMPs follow single or multiple pathways to reach their folded states; computational methods can illuminate the routes of folding and importance of structural motifs in folding and assembly; and NMR and MS may provide insights into the structures and complexes formed by OMPs. In tandem with these developments our ability to work with and manipulate membrane proteins is being augmented by the introduction of new membrane mimetics such as nanodiscs, amphipols and SMALPs [76,77]. Now is an exciting time to build on the fundamental knowledge of OMP folding and insertion gained over the last two decades and apply the full arsenal of techniques at our command to solve the problem of outer membrane protein folding.

## Abbreviations

BAM: β-barrel assembly machine
DDPC: didecanoylphosphatidylcholine
DLPC: dilauroylphosphatidylcholine
DMPC: dimyristoylphosphatidylcholine
DOPC: dioleoylphosphatidylcholine
KTSE: kinetics of tertiary structure formation by electrophoresis
LUV: large unilamellar vesicle
OM: outer membrane
OMP: outer membrane protein
SMT: single-molecule tracking
SUV: small unilamellar vesicles
